# Isoflurane and Carbon Dioxide Elicit Similar Behavioral Responses in Rats

**DOI:** 10.3390/ani10081431

**Published:** 2020-08-16

**Authors:** Satyajit Kulkarni, Debra Hickman

**Affiliations:** School of Medicine, Indiana University, Indianapolis, IN 46202, USA; kulkarni@iu.edu

**Keywords:** rat, isoflurane, carbon dioxide, aversion

## Abstract

**Simple Summary:**

Carbon dioxide and isoflurane are gases with anesthetic properties that are commonly used in laboratory rodents, especially when anesthetic overdose is used for euthanasia procedures. Concerns have been raised with the use of carbon dioxide as a euthanasia agent due to behavioral responses that indicate potential distress. This study was designed to assess aversive responses in experimentally naïve Sprague–Dawley rats when exposed to isoflurane or carbon dioxide. When placed in the forced exposure apparatus, these naïve rats were more active in the isoflurane and CO_2_ treatments compared to the control groups, suggesting that isoflurane and CO_2_ are similarly aversive. The results from the aversion-avoidance experiment supported previous work which demonstrated that while CO_2_ is more aversive than isoflurane on initial exposure, rats showed increased aversion when the isoflurane exposure was repeated. We also show that learned aversion to isoflurane is sustained for at least 15 days after initial exposure. Given this result, we suggest that CO_2_ is superior to isoflurane when euthanizing rodents with prior exposure to isoflurane. Overall, these results confirm previous studies which suggest that care should be taken when considering the serial use of isoflurane as an anesthetic.

**Abstract:**

Euthanasia in rodents is an ongoing topic of debate due to concerns regarding the aversive nature of gases with anesthetic properties such as carbon dioxide (CO_2_) and isoflurane. The aim of this study was to expand upon previously published work evaluating the aversiveness of CO_2_ by introducing an isoflurane treatment group in parallel. Aversion was tested using a forced exposure setup and an aversion-avoidance setup. In the first part of the study, 12 naïve female Sprague–Dawley rats were exposed during four consecutive days, once to each of four treatments: isoflurane, fox urine, oxygen, and CO_2_. In the second part of the study, 24 naïve female Sprague–Dawley rats and 12 rats from the first experiment were exposed to CO_2_, isoflurane, or both gases. In the forced exposure study, there were no significant differences between CO_2_ and isoflurane treatments except in line crosses. Overall, rats were more active in the isoflurane and CO_2_ treatments compared to the control groups, suggesting that isoflurane and CO_2_ are similarly aversive. In the aversion-avoidance study, rats previously exposed to isoflurane left the dark chamber significantly earlier compared to naïve rats during exposure to isoflurane. We also show that learned aversion to isoflurane is sustained for at least 15 days after initial exposure. Given this result, we suggest that CO_2_ is superior to isoflurane when euthanizing rodents with prior exposure to isoflurane. Overall, these results confirm previous studies which suggest that care should be taken when considering the serial use of isoflurane as an anesthetic.

## 1. Introduction

The appropriate use of inhalant anesthetic gases, such as carbon dioxide (CO_2_) and isoflurane, for anesthetic overdose during euthanasia in rodents is an ongoing topic of debate due to concerns regarding the aversive nature of both of these gases [1,2]. Many studies have focused on CO_2_ and have suggested the existence of negative effects of CO_2_ euthanasia [3,4,5,6,7,8,9,10,11,12,13] while others have contradicted these claims [1,11,14,15,16,17,18,19]. There is also variability in euthanasia practices by region to comply with geographic guidelines. For example, the Canadian Council on Animal Care (CCAC) recommends the use of inhalant anesthetics followed by a secondary method of euthanasia [20]. Euthanasia using CO_2_ in rodents is listed as a “conditionally acceptable method” and described as “not an ideal method of euthanasia” by the CCAC. However, when necessary, the CCAC recommends a gradual-fill rate between 20–30% chamber volume per minute. This contrasts with recent guidelines published by the American Veterinary Medical Association (AVMA) that recommends a 30–70% chamber volume per minute displacement for CO_2_ euthanasia [21].

Various studies [7,9,10,12,13] have investigated the effect of CO_2_ on rat behavior and concluded that CO_2_ is an aversive agent. This led to the argument that using halogenated inhalant anesthetics prior to the use of CO_2_ may be a refinement in euthanasia practices. Coenen et al. [12] used four methods (pre-fill, high flow rate, low flow rate, and a mixture of CO_2_ and O_2_) of CO_2_ euthanasia to determine their effects on rat behavior, brain activity, and heart rate. The authors observed high behavioral agitation and asphyxia in the pre-filled chamber condition, which is consistent with other studies [4,7,8,9,10]. They also noted lower levels of these behaviors in the high flow rate and low flow rate conditions. However, some authors [2,7] advocate against higher flow rates, so the study by Coenen et al. [12] added to the growing literature on this controversial topic of discussion. One controversial observation in this study is the absence of agitation and asphyxia with the mixture of CO_2_ and O_2_, which has been contradicted in various studies [8,15,22] since then. Another study by Niel and Weary [7] investigated the effect of CO_2_ gradual-fill of a 17.25% chamber volume displacement rate during CO_2_ euthanasia on behavior by dividing the gas exposure period into shorter segments. They observed an increase in escape behaviors and vocalizations, which are consistent with the observations by Coenen et al. [12]. The negative effects of CO_2_ exposure were also illustrated in the study by Leach et al. [9], who investigated the degree of aversion of rats and mice to CO_2_, argon, and CO_2_–argon mixtures. The authors observed higher aversion to CO_2_ and CO_2_–argon mixtures compared to argon alone based on the initial withdrawal and total dwelling times. However, they did not observe any significant difference in aversion-associated behaviors during CO_2_ exposure except for grooming behaviors.

As has been done extensively for CO_2_, studies have investigated the effect of isoflurane on behavior and reported an effect of learned aversion [13,23,24,25]. In Wong, Makowska, and Weary [23], the authors investigated total dwelling time in a light–dark apparatus. They observed that 10/16 rats stayed in the dark chamber until recumbent during initial exposure to isoflurane, but only 1/16 rats did the same during re-exposure to isoflurane. Similarly, Bertolus et al. [24] used an approach-avoidance test to investigate aversion to isoflurane and found that while 7/18 rats stayed until recumbent during initial exposure, only 1/16 stayed during re-exposure. The authors concluded that rats find isoflurane aversive during initial exposure and that this aversion increases on re-exposure to isoflurane. This is an important consideration because research animals may have been exposed to isoflurane prior to euthanasia for clinical or research purposes. Consequently, previous exposure to isoflurane could influence rat welfare during euthanasia procedures because it would be as aversive as using CO_2_ only. In this scenario, isoflurane use during euthanasia would not be a refinement over CO_2_ use during euthanasia [25]. Makowska and Weary [25] performed a similar experiment as in Bertolus et al. [24] to measure aversion to isoflurane. They observed a generalized aversion to isoflurane and halothane on re-exposure, indicating a component of learned aversion to halogenated anesthetics when previously exposed. However, the authors recommended the use of halogenated anesthetics as a refinement over CO_2_ despite the effect of learned aversion to isoflurane and their lack of a CO_2_ treatment group in parallel.

One critical limitation in these studies that investigate the possible aversive effects of CO_2_ and conclude that isoflurane is a refinement over CO_2_ during euthanasia is a lack of a head-to-head comparison between CO_2_ and isoflurane. Therefore, our aim in this study was to investigate the effect of both CO_2_ and isoflurane in the forced exposure and aversion-avoidance experimental paradigms to determine if there was a significant difference between CO_2_ and isoflurane exposure, with both previously exposed and naïve rats. In the forced exposure paradigm, we expected that there would be no significant differences in behavioral responses during CO_2_ and isoflurane exposure, and that these responses would be significantly different from control groups. Similarly, in the aversion-avoidance paradigm, we expected there would be no significant difference in the time to leave the dark chamber between CO_2_ and isoflurane, but we expected a significant difference in the time to leave the dark chamber between naïve rats and previously exposed rats.

## 2. Materials and Methods

### 2.1. Ethical Statement

All procedures were reviewed and approved by the Indiana University School of Medicine IACUC prior to initiation of the project. The program is accredited by AAALAC International and compliant with all applicable federal regulations.

### 2.2. Husbandry

As justified in Améndola and Weary [4], a sample size of 8 animals is necessary to observe treatment differences. Based on previous work in our laboratory and others, we estimated a mean behavioral relative frequency of 4.75 versus 4.25, with a standard deviation of 0.6. With a power of 0.8 and an alpha of 5%, the calculated sample size was 12 animals to account for individual differences. All rats were pair-housed in standard rat shoebox caging on an individually ventilated caging (IVC) system rack (Lab Products, Seaford, DE, USA). The cages were bedded with pine chip bedding (Sani-Chip, PJ Murphy Forest Products, Montville, NJ, USA) and nesting material (for enrichment), and each cage was changed weekly. Food (Teklad 2018SX, Envigo, Indianapolis, IN, USA) was provided without restriction. Reverse-osmosis-treated water was provided without restriction through an automatic watering system. Cages were changed at least weekly in a laminar flow workstation (NuAire, Plymouth, MN, USA) and were autoclaved prior to reuse. Hands and implements were disinfected with MB10 (Quip Labs, Wilmington, DE, USA) between cages. The macroenvironment was on a 12:12-h light:dark cycle (lights on, 07:00 h), and the temperature was maintained at 72 ± 1 °F (22.2 °C ± 0.5 °C), with humidity maintained between 30% and 70%. The colony was screened quarterly by using indirect sentinels. At the time of the study, the colony was free of the following pathogens: coronavirus (sialodacryoadenitis virus), parvoviruses (NS1, rat pneumonia virus, Kilham rat virus, H1 virus, rat minute virus), theliovirus, *Clostridium piliforme*, *Mycoplasma pulmonis*, pinworms (*Aspicularis tetraptera*, *Syphacia* spp.), and fur mites (*Radfordia ensifer*, *Ornithonyssus bacoti*).

### 2.3. Experiment 1: Forced Exposure

#### 2.3.1. Apparatus

**Figure 1:**
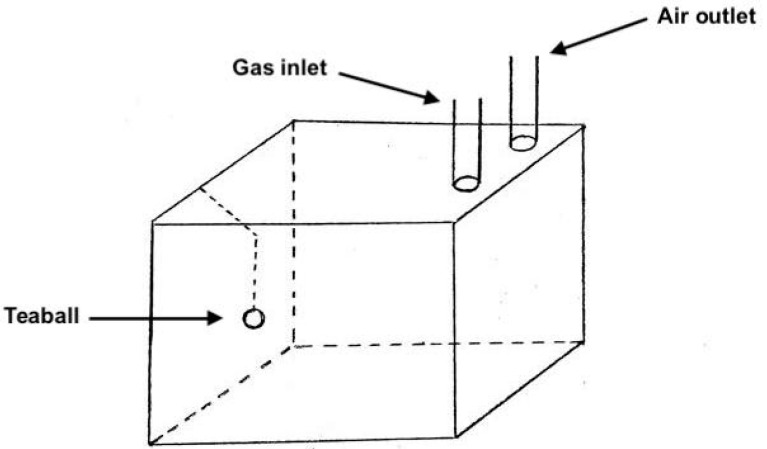
Schematic of the chamber used for the forced exposure experiment.

All trials were performed in an acrylic glass cage (23 × 45 × 24 cm/24.84 L) with aspen chip bedding (Teklad 7090, Envigo, Indianapolis, IN, USA). The cage was covered with an acrylic glass lid that contained a gas inlet and an air outlet on the right side (11.25 × 5.75 cm from the corner of the box, 11.5 cm from each other), and a metallic tea ball (3 in. diameter) on the left side (Figure 1). CO_2_ was delivered into the cage using compressed gas cylinders through a clear vinyl tube inserted in the gas inlet. A flow meter connected to the compressed cylinder was used to regulate gas flow. Isoflurane and oxygen were delivered using a precision vaporizer through a clear vinyl tube inserted in the gas inlet. Passive scavenging was used. Gas concentrations were not monitored to confirm distribution.

#### 2.3.2. Experimental Design

As in Améndola and Weary [4], twelve adult (ranging from 180 to 217 days of age) female Sprague–Dawley rats were exposed during four consecutive days, once to each of four treatments: isoflurane gradual-fill (3% in O_2_; 14% chamber vol. min^−1^), CO_2_ gradual-fill (18.5% chamber vol. min^−1^), oxygen gradual-fill (O_2_; 14% chamber vol. min^−1^; control), and fox scent (5 μL fox urine scent placed on a tissue paper and placed in a 3 in. diameter tea ball; passive response eliciting stimulus). Our rationale in using 3% isoflurane gradual-fill in O_2_ (14% chamber vol. min^−1^) was to enable exposure for the full testing period without the rats becoming unconscious, and is in line with previous studies [26,27,28]. These rats were first generation offspring produced by breeder pairs of Sprague–Dawley rats (Crl:CD; Charles River Laboratories, Wilmington, MA, USA). The breeder pairs and their offspring were raised under variable light conditions for a separate study. Pups were removed from the variable light conditions upon weaning (approximately 21 to 25 days of age) and were housed with no further experimental manipulation until commencement of this study. Each rat in each group was individually marked (1, 2, 3, or 4) on the tail with a permanent marker based on the initial order of exposure. Order of exposure was allocated using three 4 × 4 Latin squares (four rats and four treatments: CO_2_, oxygen, isoflurane, and fox scent). Three days after exposure to all four treatments, the same twelve rats were re-exposed to the treatments, allocating treatment order in three different 4 × 4 Latin squares (Table S1).

#### 2.3.3. Testing Procedure

As in Améndola and Weary [4], rats were individually placed in the experimental cage covered with the lid and remained there for 5 min to acclimate. Following this period, the rat was exposed to the treatment for 2 min. For the fox scent treatment, the tea ball containing filter paper with 5 μL of fox scent was attached during the experimental period. For CO_2_, isoflurane, and oxygen treatments, the tea ball attached during the experimental period was empty. After the tea ball was in place, the gas flow was started. Tests were stopped when the animal became ataxic or after 2 min, whichever came first. Ataxia was defined as when the animal started to have difficulty walking and started to fall on its side (but could still right itself) and was always identified by the same observer. Our intention was to stop before the animal reached the loss-of-righting-reflex stage. After each test, the experimental cage and lids were cleaned with Rescue (Virox Animal Health, Oakville, OH, USA) and bedding was replaced. All trials were performed under white light between 12:00 and 16:00 h.

#### 2.3.4. Behavioral Observations

Forced exposure trials were video recorded. As in Améndola and Weary [4], the videos were divided into baseline (60 s before any test) and active periods (first 60 s of the test). These two periods were scored for a total of 120 s. The frequency of active and passive behaviors (Table 1) were recorded by an observer who was blinded to treatment. Total time spent on either side of the experimental cage (tea ball or gas inlet) was also recorded.

#### 2.3.5. Expected Outcomes

We hypothesized that we would observe statistically significant increases in active behaviors for the rats exposed to CO_2_ and isoflurane, but not oxygen or fox urine. Minimal response was expected with fox urine based on Améndola and Weary [4]. We also hypothesized that there would be no significant difference in the active and passive behaviors between the CO_2_ and isoflurane groups.

### 2.4. Experiment 2: Aversion-Avoidance

#### 2.4.1. Apparatus

**Figure 2:**
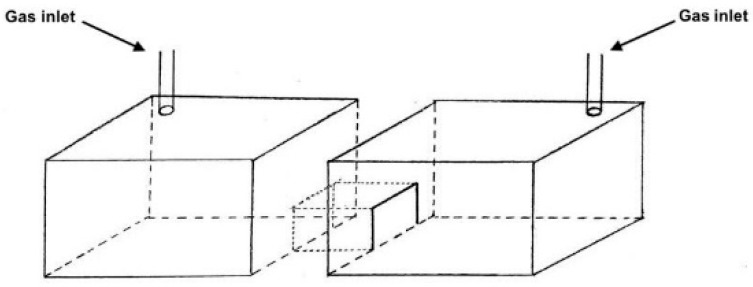
Schematic of the chamber used for the aversion-avoidance experiment.

The apparatus consisted of a plastic light–dark box consisting of two compartments (43.2 × 34.0 × 19.8 cm each), connected by a small red buffer compartment (10 × 14 × 30 cm). The light compartment was covered with white plastic on the sides and illuminated by two bulbs placed above the lid. The light intensity was measured to be 3920 Lux at the bottom of the compartment. The dark compartment was covered with a layer of red plastic and a layer of black plastic. The light intensity was measured to be 85 Lux at the bottom of the compartment. Both compartments contained bedding. Doorways of the buffer compartment were covered with black plastic flaps. The light and dark compartments of the light-dark box were covered with plastic lids. The lid contained a gas inlet in the upper-left and upper-right corner of the light and dark compartments (10.8 × 8.5 cm from the corner of the box) respectively as seen in Figure 2. The lid corresponding to the dark compartment was covered with a layer of red plastic and a layer of black plastic. Oxygen and isoflurane flow were regulated using a flow meter integrated in an anesthetic machine. CO_2_ flow was regulated using a flow meter and delivered from a compressed gas cylinder. All gases were delivered through a clear vinyl tube inserted in the gas inlets. Passive scavenging was used. Gas concentrations were not monitored to confirm distribution.

#### 2.4.2. Habituation and Training

Rats were habituated to the light–dark box over two consecutive days. Each subject was placed in the light compartment of the apparatus and left to explore for 30 min. On the second day, oxygen gas (12% chamber vol. min^−1^) was delivered in both compartments.

#### 2.4.3. Experimental Design

A total of 24 naïve female Sprague–Dawley rats and 12 rats from Experiment 1 were used (total *n* = 36). One group of naïve rats (*n* = 12) was exposed twice to CO_2_ gradual-fill (approximately 19% displacement of chamber volume min^−1^) over two consecutive days. A second group of naïve rats (*n* = 12) was exposed twice to isoflurane gradual-fill (3% in O_2_; 12% chamber vol. min^−1^) over two consecutive days. A third group of rats (*n* = 6) from Experiment 1 was exposed twice to isoflurane gradual-fill (3% in O_2_; 12% chamber vol. min^−1^) over two consecutive days and then twice to CO_2_ gradual-fill (approximately 19% displacement of chamber volume min^−1^) over two consecutive days. A fourth group of rats (*n* = 6) from Experiment 1 was exposed twice to CO_2_ gradual-fill (approximately 19% displacement of chamber volume min^−1^) over two consecutive days and then twice to isoflurane gradual-fill (3% in O_2_; 12% chamber vol. min^−1^) over two consecutive days (Table S2).

#### 2.4.4. Testing Procedure

As in Améndola and Weary [4], rats were individually placed in the light compartment of the light–dark box and allowed to explore the apparatus for 30 min with oxygen gas (12% chamber vol. min^−1^) flowing to both compartments. We anticipated that all subjects would settle down in the dark compartment for at least 10 min by the end of the 30 min acclimation period. CO_2_ or isoflurane flow was then started in the dark compartment depending on the treatment group. The test was stopped when the rat indicated intention to move into the light compartment (i.e., both shoulders crossed from the dark compartment into the buffer compartment) or if the rat was not recumbent by 300 s from the start of gas flow. No specific steps were taken to randomize the order of testing. The data were collected over time and at least two different treatment groups were initiated on each day to create pseudo randomization. The time to leave the dark chamber (in seconds) was recorded as the dependent variable. The light–dark box was disinfected using Rescue and the bedding was replaced after each trial.

#### 2.4.5. Expected Outcomes

The time to leave the dark chamber was compared between treatment groups. We hypothesized that there would be no significant differences in the time to leave the dark chamber between the isoflurane and CO_2_ treatment groups. We also expected a significant difference in time to leave the dark chamber between the naïve rats and rats that had previous exposure to isoflurane and CO_2_.

### 2.5. Data Analysis

Analysis was performed using JMP Pro 15 software (SAS, Cary, NC, USA).

#### 2.5.1. Analysis for Experiment 1: Forced Exposure

As in Améndola and Weary [4], rat responses were compared between treatment groups. Two behaviors (nose touches and bedding manipulations) were not analyzed due to their infrequency, which greatly skewed the data and increased the possibility of erroneous findings. Relative frequencies of active and passive behaviors were used as response variables. Relative frequency of behaviors was calculated by dividing the frequency of the behavior by time scored. Three models were used to compare these rat responses: baseline, active, and change from baseline. Within each model, the effect of treatment group and day of exposure (exposure vs. re-exposure) was investigated. The baseline data between the conditions were compared between the treatments to ensure that there was no significant difference as the rats were not being exposed to any treatment. The active data were compared between the treatments to identify the effect of treatment on the behaviors. Change from baseline (relative frequency of behavior in active period − relative frequency of behavior in baseline period) was also investigated for each behavior to compare results with Améndola and Weary [4]. First, normality of data and homogeneity of variance was determined using the Anderson–Darling test and Levene’s test, respectively. For the baseline data, homogeneity of variance was satisfied but the data were not normally distributed. For this reason, the Kruskal–Wallis test was applied to the baseline data. For the active and change from baseline data, both normality of data and homogeneity of variance were not satisfied, so Welch’s ANOVA was applied to investigate overall effects. Post-hoc Games–Howell analysis was performed to investigate specific differences between groups.

#### 2.5.2. Analysis for Experiment 2: Aversion-Avoidance

Three models were used with time to leave the dark chamber as the response variable. The first model compared the effect of treatment group (previously exposed vs. naïve), treatment (CO_2_ vs. isoflurane), and the interaction between treatment group and treatment. The second model investigated the effect of order of exposure (i.e., CO_2_ first, isoflurane second vs. isoflurane first, CO_2_ s) in the previously exposed group. The third model investigated the effect of day of exposure (exposure vs. re-exposure) in the naïve group for isoflurane and CO_2_. The third model was not applied to data from the forced exposure group because they had been previously exposed to both isoflurane and CO_2_. First, normality of data and homogeneity of variance was determined using the Anderson–Darling test and Levene’s test, respectively. Homogeneity of variance was satisfied for the CO_2_ treatment group but not for the isoflurane treatment group. The data were also not normally distributed. The data were further examined by computing the residuals for each group (FE/CO_2_; FE/isoflurane; Naïve/CO_2_; Naïve/isoflurane) and testing normality of the residuals and homogeneity of variance of the residuals. Residuals were calculated by subtracting the mean of the response variable for each group from each observation within the group. Homogeneity of variance was satisfied for the residuals of the CO_2_ treatment group but not for the isoflurane treatment group. The residual data were also not normally distributed. A natural log (ln) transformation was performed to make the data fit the assumptions of normality of data and homogeneity of variance. As before, normality of data and homogeneity of variance was determined using the Anderson–Darling test and Levene’s test, respectively. Both assumptions were satisfied in the ln-transformed data. Normality of data and homogeneity of variance were also satisfied for the residuals of the ln-transformed data. Repeated measures ANOVA (standard least squares with effect screening) was performed on the ln-transformed data to investigate overall effects as there were two nominal variables. A one-way ANOVA was performed to investigate the effect of treatment group (previously exposed vs. naïve) within each treatment (CO_2_ vs. isoflurane).

## 3. Results

### 3.1. Forced Exposure

#### 3.1.1. Baseline Data

There were no significant differences between treatment groups for rears (degrees of freedom (df) = 3; H = 0.2971; *p* = 0.9606), line crosses (df = 3; H = 1.9967; *p* = 0.5731), time immobile (df = 3; H = 0.7877; *p* = 0.8524), time by gas inlet (df = 3; H = 1.8928; *p* = 0.5950), and time by tea ball (df = 3; H = 1.8928; *p* = 0.5950).

#### 3.1.2. Active Data

The active behavior data are summarized in the table below (Table 2). For rears, there was a statistically significant difference between treatment groups (Table 2). Post-hoc analysis showed a significant difference between CO_2_ and fox urine (*p* = 0.0331), isoflurane and fox urine (*p* = 0.0043), and isoflurane and oxygen (*p* = 0.0304). On initial exposure, there was no significant difference between treatment groups (df = 3, 22.795; F = 1.2221; *p* = 0.3244), but there was a significant difference between treatment groups on re-exposure (df = 3, 23.399; F = 6.8836; *p* = 0.0017). Post-hoc analysis showed a significant difference between CO_2_ and fox urine (*p* = 0.0216) and isoflurane and fox urine (*p* = 0.0037) on re-exposure.

For line crosses, there was a statistically significant difference between treatment groups (Table 2). Post-hoc analysis showed a significant difference between CO_2_ and oxygen (*p* = 0.0003), CO_2_ and fox urine (*p* = 0.0027), and CO_2_ and isoflurane (*p* = 0.0149). There was a significant difference between groups on initial exposure (df = 3, 23.023; F = 5.3925; *p* = 0.0058) and on re-exposure (df = 3, 23.75; F = 4.0182; *p* = 0.018). Post-hoc analysis showed a statistically significant difference between CO_2_ and oxygen (*p* = 0.0047) on initial exposure, and CO_2_ and fox urine (*p* = 0.0126) on re-exposure.

For immobility time, there was a statistically significant difference between treatment groups (Table 2). Post-hoc analysis did not show a statistically significant difference between CO_2_ and oxygen (*p* = 0.0780), and isoflurane and oxygen (*p* = 0.0757). There was no significant difference between groups on initial exposure (df = 3, 23.638; F = 0.5655; *p* = 0.6431), but there was a significant difference between groups on re-exposure (df = 3, 23.014; F = 3.9085; *p* = 0.0216). Post-hoc analysis showed a significant difference between CO_2_ and oxygen (*p* = 0.0330) on re-exposure. There was no significant difference between treatment groups (Table 2) for time spent by the gas inlet and for time spent by the tea ball.

#### 3.1.3. Change from Baseline Data

The change from baseline data are summarized in the table below (Table 3). For rears, there was a statistically significant effect of treatment group (Table 3). Post-hoc analysis did not show any significant difference between groups on initial exposure, but there was a significant difference between CO_2_ and fox urine (*p* = 0.0492) and isoflurane and fox urine (*p* = 0.0154) on re-exposure. For line crosses, there was no statistically significant effect of treatment group (Table 3). Post-hoc analysis did not show any significant difference between groups on initial exposure, but there was a significant difference between CO_2_ and fox urine (*p* = 0.0319) on re-exposure.

For immobility time, there was no statistically significant difference between treatment groups (Table 3). Post-hoc analysis did not show a significant difference between treatment groups on initial exposure or re-exposure. For time by gas inlet, there was no significant difference between treatment groups (Table 3). Post-hoc analysis did not show a significant difference between groups on initial exposure or re-exposure. For time by tea ball, there was no significant difference between treatment groups (Table 3). Post-hoc analysis did not show a significant difference between groups on initial exposure or re-exposure.

### 3.2. Aversion-Avoidance

The data in Table 4 show an overall statistical significance in time to leave the dark chamber between the treatment groups and previous exposure to the gases (df = 3, 91; F = 4.6531; *p* = 0.0045). There was no significant interaction of the treatment group and whether the animals had been previously exposed to CO_2_/isoflurane (i.e., animals from the forced exposure experiment) on time to leave the dark chamber (Table 4). There was a statistically significant difference between the forced exposure groups (meaning they had been previously exposed to both isoflurane and CO_2_) and the naïve group (Table 4). The naïve group (Mean: 103.3 s; 95% CI: 82.9 to 128.6) stayed in the dark chamber for a longer time compared to the forced exposure group (Mean: 69.72 s; 95% CI: 56.0 to 87.2) overall. There was also a statistically significant effect of the treatment group on time to leave the dark chamber (Table 4). Animals in the CO_2_ group (Mean: 69.42 s; 95% CI: 55.7 to 86.4) stayed in the dark chamber for a shorter time than the animals in the isoflurane group (Mean: 104.64 s; 95% CI: 83.3 to 129.8) overall. In the forced exposure group, there was no statistically significant effect of order of exposure (CO_2_ first vs. isoflurane first) on time to leave the dark chamber when exposed to CO_2_ (df = 1, 22; F = 0.1012; *p* = 0.7534) or isoflurane (df = 1, 21; F = 2.0309; *p* = 0.1688). Within the naïve group, there was no statistically significant effect of day of exposure on time to leave the dark chamber for isoflurane (df = 1, 22; F = 0.3883; *p* = 0.5396) or CO_2_ (df = 1, 22; F = 0.0169; *p* = 0.8978). There was no significant difference in time to leave the dark chamber between the CO_2_ and isoflurane exposures for rats previously exposed to both agents (df = 1, 45; F = 1.0675; *p* = 0.3070).

**Figure 3:**
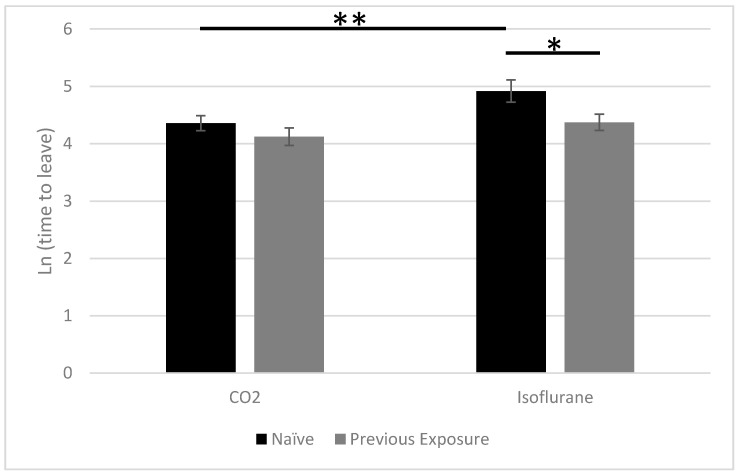
Naïve rats (**dark bar**) vs. previously exposed rats (**light bar**) in natural log time to leave the dark chamber when comparing within treatment groups. An asterisk (* or **) signifies when the *p* value is significant (*p* < 0.05).

The data were further analyzed within each treatment (CO_2_ vs. isoflurane) and group (forced exposure vs. naïve) using a one-way ANOVA (Figure 3). There was no statistically significant difference (df = 1, 46; F = 1.0160; *p* = 0.3187) in time to leave the dark chamber between the forced exposure group and naïve group when exposed to CO_2_. When exposed to isoflurane, there was a statistically significant difference in time to leave the dark chamber between the forced exposure group and the naïve group (df = 1, 45; F = 6.8986; *p* = 0.0117). There was no significant difference in time to leave the dark chamber between the CO_2_ and isoflurane exposures for rats previously exposed to both agents (df = 1, 45; F = 1.0675; *p* = 0.3070). However, there was a significant difference in time to leave the dark chamber between the CO_2_ and isoflurane exposures for naïve rats (df = 1, 46; F = 7.8289; *p* = 0.0075).

## 4. Discussion

### 4.1. Forced Exposure

#### 4.1.1. Baseline Data

We did not expect any significant differences between the treatment groups during this period because the rats were not being exposed to any treatment. Our data match this prediction.

#### 4.1.2. Active Data

For the active period, we expected that the isoflurane and CO_2_ treatments would show significantly higher relative frequencies of active behaviors when compared with the fox urine and oxygen treatments. For rears, we observed a significant increase when comparing CO_2_ and fox urine, isoflurane and fox urine, and isoflurane and oxygen. This matches with our initial prediction. A significant increase in rearing during CO_2_ exposure compared to the control group has been previously observed by Niel and Weary [7], but other studies [8,9,10] have failed to observe this trend. Moreover, our results show a significant increase in rearing during isoflurane exposure compared to the control groups, which has not been observed in previous studies [8,10]. This increase was significant during re-exposure between CO_2_ and fox urine, and isoflurane and fox urine. This indicates that both isoflurane and CO_2_ elicit an increase in rearing during re-exposure, which could be interpreted as an attempt to escape gas exposure.

For line crosses, we observed a significant increase when comparing CO_2_ with oxygen, isoflurane, and fox urine. This increase was significant between the CO_2_ group and the control groups during both initial exposure and re-exposure. These results are consistent with a previous study [7] that found an increase in line crossing for CO_2_ when compared to air. Like rearing, this could be interpreted as an attempt to escape gas exposure in rats exposed to CO_2_. Clearly, in this case, CO_2_ elicited a different behavioral response when compared to isoflurane.

Moreover, there was a significant decrease in immobility time during re-exposure when comparing CO_2_ and oxygen. Rats exposed to CO_2_ and isoflurane spent more time compared to the control groups in active behaviors such as rearing and line crossing, so it logically follows that there would be a difference in immobility time between groups. The increase in active behaviors for the CO_2_ and isoflurane groups was expected because the gases cause agitation and can be interpreted as an effort by the animal to escape the gas. An increase in active behaviors would mean less time was spent immobile, which also is consistent with the data as there was a significant difference in immobility time between the groups. An interesting behavioral observation is that there was no significant difference in the time spent on either side of the apparatus. It was expected that the animals would spend less time near the source of the gas if it were aversive. However, it is possible that they could not escape the gas within the chamber as the apparatus was small, which could have led to the gas distributing equally in the chamber during the exposure period. Thus, there was no advantage to being on either side of the chamber, and the animals tried to actively escape the aversive stimulus by searching for escape routes on either side of the cage, leading to equal time spent on either side of the cage. We did not measure the distribution of gases due to the logistical issues with measuring distribution in the fox urine and isoflurane treatments, but movement of the animal would have caused the gas to distribute equally in the apparatus during the exposure period.

#### 4.1.3. Change from Baseline Data

For rears, there was a significant effect of treatment group. The relative frequency of rears was significantly higher during re-exposure between CO_2_ and fox urine, and isoflurane and fox urine. This difference between CO_2_ and fox urine on re-exposure is consistent with the data from Améndola and Weary [4]. However, Améndola and Weary [4] observed this difference during initial exposure to CO_2_ as well, which was not observed in this study. Possible reasons for the differences between this study and Améndola and Weary [4] are discussed later in the Comparisons section of the discussion.

For line crosses, we did not observe a significant effect of treatment group. We did not observe any difference between groups on initial exposure, but there was a significant increase in line crosses between CO_2_ and fox urine on re-exposure. While the latter statement is consistent with results from Améndola and Weary [4], the former is not. Améndola and Weary [4] also observed an overall significant effect of treatment group on line crossing behavior, which was not observed in this study.

For immobility time, we did not observe a significant effect of treatment group. We did not observe any effect of exposure number (exposure vs. re-exposure) on immobility time between treatment groups either. Again, this result conflicts with data from Améndola and Weary [4], who observed a significant difference in immobility time between CO_2_ and fox urine, and CO_2_ and oxygen on re-exposure. There was a large standard error in this behavior, which could be attributed to individual variation within groups. This indicates that there is individual variation in sensitivity when exposed to CO_2_, fox urine, isoflurane, and oxygen, as evidenced by Améndola and Weary [4].

There was no significant difference in time spent on either side of the apparatus for any of the treatment groups. Again, there was high variation for this variable, and this could be attributed to individual differences between the animals. This result suggests that the rats did not attempt to actively avoid the location for the entry point of the anesthetic/control agent. As discussed previously, this could be because the small size of the chamber and because the movement of the rat enabled equal distribution of the gas in the chamber, thus creating no advantage of being away from the gas inlet.

### 4.2. Aversion-Avoidance

#### 4.2.1. Between Treatment Groups

In the aversion-avoidance experiment, there was an overall significant effect of treatment group and previous exposure to the gases on the time to leave the dark chamber. Rats with previous exposure to CO_2_ and isoflurane left the dark chamber significantly earlier when compared to naïve rats. This finding suggests that previous exposure to anesthetic gases can lead to increased aversion during re-exposure. Moreover, there was a significant effect of treatment group on time to leave the dark chamber. Rats in the CO_2_ group stayed in the dark chamber for a shorter time when compared to the isoflurane group. This finding indicates that CO_2_ is possibly more aversive to rats than isoflurane overall. There is a stark difference in the average time to leave the dark chamber during CO_2_ exposure when comparing the results of this study with those obtained by Améndola and Weary [4]. The rats in their study (which had been previously exposed in their forced exposure experiment) stayed in the dark chamber for an average of 35 s during initial exposure and 33 s on re-exposure. On the other hand, the rats in our study stayed in the dark chamber for an average of 61.67 s when previously exposed and 79.25 s when naïve. The possible reasons for this difference are discussed later in the Comparisons section of the discussion.

#### 4.2.2. Within Treatment Groups

Rats in the CO_2_ group left the dark chamber earlier than the rats in the isoflurane group when the animals were naïve to both gases. However, there was no significant difference in the time to leave the dark chamber between CO_2_ and isoflurane for the rats with previous exposure to the gases. This is consistent with a recent study [13] which showed that isoflurane and CO_2_ are similarly aversive to mice. Based on this data, the use of isoflurane as an anesthetic prior to euthanasia with CO_2_ is not a refinement if the rats have been previously exposed to isoflurane. In fact, some studies [23,25] have shown that there is a generalized learned aversion to halogenated anesthetics (e.g., isoflurane, halothane) in rats, which means that prior use of halogenated anesthetics could result in distress to the animal if isoflurane is used during euthanasia procedures. The mechanism by which this generalized aversion develops is not well-characterized, and it is also not known if this generalized aversion is sustained in the days after exposure to halogenated anesthetics.

Furthermore, within the CO_2_ group, there was no significant difference between rats previously exposed to CO_2_ and rats that were naïve to CO_2_. This suggests that CO_2_ was not more aversive on re-exposure. However, within the isoflurane group, naïve rats stayed in the dark chamber for significantly longer when compared to rats previously exposed to isoflurane. This is consistent with previous studies that indicate a component of learned aversion in exposure to isoflurane [24,25]. Some researchers [13,25] contend that this aversion to isoflurane is transient, and is not sustained in the days after exposure, thus eliminating the idea that use of isoflurane during procedures requiring anesthesia has an impact on the use of isoflurane during euthanasia. However, a recent study [29] found this learned aversion to isoflurane is maintained for up to 7 days after initial exposure to isoflurane. Our study builds on this data. We re-exposed rats from the forced exposure experiment to isoflurane during the aversion-avoidance experiment at least 15 days after their first exposure to isoflurane in the former experiment. Our results found that rats that had been previously exposed to isoflurane left the dark chamber significantly earlier when compared to naïve rats during exposure to isoflurane. If the effects of learned aversion were transient, there would not be a difference between these two groups. Thus, our study shows that aversion to isoflurane is sustained for at least 15 days after initial exposure to isoflurane. Given this finding, we would not recommend the use of isoflurane for euthanasia if there has been prior exposure of the rat to isoflurane. Repeat use of isoflurane should probably also be avoided, if possible, given the sustained aversion associated with this anesthetic agent, though the length of time that this learned aversion is sustained is worthy of an additional study.

Moreover, while the data did not show a statistically significant effect of day of exposure (exposure vs. re-exposure) on latency to leave the dark chamber in naïve rats, it is possible that the rats did not fully acclimate to this treatment as illustrated by the learned aversion to isoflurane in the forced exposure group. The lack of a statistically significant effect of day of exposure on time to leave the dark chamber for naïve rats when exposed to CO_2_ is consistent with results from Améndola and Weary [4].

### 4.3. Comparisons

There are several ideas that could explain the differences in results between this study and the study performed by Améndola and Weary [4]. Firstly, the stock/strain difference could account for the difference in sensitivity to the agents used [1,30,31,32,33,34]. This study used first generation offspring from Sprague–Dawley rats obtained from Charles River laboratories, while Améndola and Weary [4] used surplus Sprague–Dawley animals from the University of British Columbia, but did not provide information regarding the original source of these animals. Previous studies [35,36] have shown that there is an effect of sub-strain (vendor) on rodent behavior. Sub-strains of C57BL/6 mice from Charles River Laboratories (C57BL/6Crl) and Jackson Laboratory (C57BL/6J) differ significantly in their alcohol consumption behaviors. C57BL/6J mice show a significantly higher ethanol preference compared to C57BL/6Crl mice [35], but C57BL/6Crl mice show a robust ethanol deprivation effect (EDE) that is not observed in C57BL/6J mice [36]. A careful understanding of the source of the animals used in an experiment is critical to the validity of the claims made because of the experiment, due to the effect that genetic drift and differences in microbiome, among other biological differences, could have on rodent behavior. Therefore, it is possible that vendor sub-strain differences contributed to the discrepancies in the results between our study and that performed by Améndola and Weary [4]. Studies [30,34] have also shown an effect of strain on rodent behavior. Britt [34] demonstrated a difference in response to CO_2_ exposure between Sprague–Dawley and Lister-Hooded rats. Sprague–Dawley rats tended to become more active as CO_2_ concentrations increased while Lister-Hooded rats tended to spend more time immobile. Moreover, Creamer-Hente et al. [30] evaluated four strains of male and female mice and their stress responses to CO_2_ euthanasia. They observed a statistically significant effect of strain on duration of ataxia and labored breathing. These studies suggest that strain identity could play an important role on behaviors associated with aversion or effort to escape such as rearing, grooming, and line crossing. It is necessary to consider the effect of strain and sub-strain when making blanket claims on the effects of CO_2_ exposure in rodents. Consequently, tailoring of euthanasia guidelines depending on these factors may be necessary.

Additionally, previous exposure to bleach was a confounding variable in the study by Améndola and Weary [4] that could explain the differences in sensitivity to treatments. The dose and length of time that the rats were exposed to bleach were not provided, and it is known that bleach is an irritant that can cause damage to the nasal mucosa [37]. It is possible that the responses between the two studies differed because of the previous experimental manipulation of the rats in Améndola and Weary [4].

Thirdly, even though both studies coded for the same behaviors in the forced exposure experiment, there could be variation in each individual scorer and what they perceived each action as, for example, a rear. Makowska and Weary [38] discuss a lack of consistency between scorers in observations of gross behavior. Even though the definitions for behaviors scored were identical between our studies, it is possible that borderline cases (where, for example, the behavior could be perceived as a rear or not) could have differed between the observers. This perceptual difference is evidenced in Améndola and Weary’s [4] score for inter-observer reliability, which was low for some behaviors such as line crosses (r = 0.77) and bedding manipulations (r = 0.76), but higher for other behaviors such as rears (r = 0.91) and immobility time (r = 0.99). It is important to note here that these scores for inter-observer reliability are based on a small sample of the videos, and measuring inter-observer reliability between two observers for all the videos could have led to lower r values. Future research could investigate differences between observers when scoring the same behaviors based on definitions.

For the aversion-avoidance study, one difference between the two studies was in the definition of the endpoint for when the rat “left” the dark compartment. Améndola and Weary [4] defined their end-point for the aversion-avoidance experiment as when the rat crossed from the buffer compartment to the light compartment. However, this study defined the end-point as when the rat crossed from the dark compartment to the buffer compartment. Therefore, it would be expected that the time to leave the dark chamber would be shorter in this study as compared to the study by Améndola and Weary [4]. Crossing from the dark compartment into the buffer compartment indicated an intention to leave the compartment. Because the buffer compartment had flaps, the rat could have settled in the buffer compartment after leaving the dark compartment if the gas was not able to pass through the buffer compartment, which is why we selected this endpoint, even though it is more conservative than the previous study.

Moreover, there is a difference in light intensity in the light compartment between our study and Améndola and Weary [4]. Their study used a light intensity of 1650 Lux while our study used a light intensity of 3920 Lux. This difference in light intensity could explain the difference in the time to leave the dark chamber values between the two studies. Lastly, the rats in the Améndola and Weary [4] study were previously exposed to CO_2_ in the forced exposure paradigm, which could have confounded their results in the following approach-avoidance and aversion-avoidance studies because the rats were no longer naïve [23].

### 4.4. Limitations and Future Research

One possible limitation in this study is that the ethogram scored was not as extensive as it could have been. Behaviors like freezing, gasping, shaking, and recoil from sniffing of the gas tube are possible behaviors that could be included in future studies investigating animal welfare focused effects of CO_2_ and various halogenated anesthetics (such as isoflurane), though there is still much discussion on which behaviors are the most relevant [39]. Behavioral assessment of stress responses is strengthened with the inclusion of physiologic data, such as evaluation of the neuroendocrine response [1,2].

Another possible limitation is that a male experimenter was present in sight and smell of the animals used in this experiment, which has been shown [40] to cause physiological stress responses in rodents. This could have been a possible confounding effect in this study. There was also no formal randomization of testing in the aversion-avoidance experiment, which could have introduced bias. Moreover, our study and the study by Améndola and Weary [4] did not measure gas concentration in various parts of the apparatus to confirm equal distribution. This could be a possible limitation in both studies as it could be a confounding effect in the design of the study or in the interpretation of data.

Another limitation in both studies is the difference in the chamber volume displacement rates between the different gases in both experiments (forced exposure and aversion-avoidance). In the forced exposure experiment, we used a 14% chamber vol. min^−1^ displacement rate for isoflurane and oxygen, and an 18.5% chamber vol. min^−1^ displacement rate for CO_2_. Améndola and Weary [4] used a 16.2% chamber vol. min^−1^ displacement rate for oxygen and an 18.5% chamber vol. min^−1^ displacement rate for CO_2_. Similarly, in the aversion-avoidance experiment, we used a 12% chamber vol. min^−1^ displacement rate for isoflurane and oxygen, and a 19% chamber vol. min^-1^ displacement rate for CO_2_. Améndola and Weary [4] used a 30.9% chamber vol. min^−1^ displacement rate for oxygen, and a 19% chamber vol. min^−1^ displacement rate for CO_2_. The differences in the volume displacement rates between groups and during habituation is a confounding effect in both studies that could have impacted the dependent variables.

Future studies could investigate the intrinsic mechanisms through which learned aversion to isoflurane is developed and the generalization of this aversion to other halogenated anesthetics like halothane [25]. It is known that CO_2_ exposure causes hypercapnia (an increase in blood CO_2_ concentration) and acidosis (a decrease in blood pH), and that the human body responds to oppose this change [41]. However, at a certain point, these responses are not sufficient and consequently, may lead to the development of negative emotional states such as air hunger [42]. It is thought that this is the primary cause of aversion to CO_2_ exposure in naïve rats. On the other hand, the mechanism of action for isoflurane is not well-characterized. It is speculated that inhalant anesthetics like isoflurane act by altering the activity of neuronal ion channels [43,44,45]. A recent study [46] found that isoflurane and sevoflurane induced hyperglycemia in adult mice but hypoglycemia in neonatal mice. This is consistent with other studies that have reported hyperglycemia in adult humans during induction with isoflurane [47] and hypoglycemia and metabolic acidosis in neonatal mice [48]. These effects could be linked to isoflurane’s mechanism of action.

Another important consideration in the clinical effect of anesthesia is that aversion during repeat exposure to inhalant anesthetics involves a component of the animal’s experience during recovery while aversion during initial exposure involves only the initial experience to the inhalant anesthetic. For this reason, development of learned aversion may stem from the recovery process due to repeat exposure to inhalant anesthetics and thus an understanding of the mechanism of action of halogenated anesthetics such as isoflurane is necessary if there is an intention to attenuate the effect of learned aversion.

## 5. Conclusions

When placed in the forced exposure apparatus, rats were more active in the isoflurane and CO_2_ treatments compared to the control groups, suggesting that isoflurane and CO_2_ are similarly aversive. The results from the aversion-avoidance apparatus supported previous work which demonstrated that while CO_2_ is more aversive than isoflurane on initial exposure, rats demonstrate increased aversion upon re-exposure to isoflurane. This effect of learned aversion is sustained for at least 15 days after initial exposure to isoflurane. Given this result, we suggest that CO_2_ is superior to isoflurane when euthanizing rodents with prior exposure to isoflurane. Overall, these results confirm previous studies [25] which suggest that care should be taken when considering serial use of isoflurane as an anesthetic.

## Figures and Tables

**Table 1:** Descriptions of the behaviors assessed in Experiment 1 (Forced Exposure).

**Active Behaviors**	Rearing: raising the upper body on the hind limbs in a vertical position with both front paws off the ground (frequency) Line-crossing: Horizontal locomotor activity that results in the rat’s forepaws crossing a line that divides the length of the chamber in half (frequency) Lid-pushing: Push at the cage lid with nose or front paws (frequency) Bedding manipulation: Displacement (pushing, shoveling, flicking, or digging) of bedding material with front and/or back paws (frequency) Grooming: Self-grooming using front paws to rub the face or ears (frequency) Nose-touching: Aggressively sniffing the air outlet or the corners of the testing apparatus (frequency)
**Passive Behaviors**	Immobility time: Absence of movement, except for small and slow lateral movements of the head (seconds)
**Other Measurements**	Time spent on either side of the cage (seconds)

**Table 2:** Data from the active period (during exposure to treatment). F ratios and *p* values are reported for main effects with significance set at *p* < 0.05. An asterisk (*) signifies when the *p* value is significant. Data are reported as mean relative frequency ± standard deviation [95% CI].

Behaviors	F Ratio [df]; *p* Value	Mean Relative Frequency ± Standard Deviation [95% CI]			
		CO_2_	Fox Urine	Isoflurane	Oxygen
Rears	6.2068 [3, 49.07]; 0.0012 *	0.109 ± 0.074 [0.077, 0.140]	0.057 ± 0.044 [0.037, 0.077]	0.102 ± 0.038 [0.085, 0.118]	0.064 ± 0.051 [0.042, 0.086]
Line crosses	7.1582 [3, 49.494]; 0.0004 *	0.057 ± 0.032 [0.044, 0.071]	0.025 ± 0.026 [0.013, 0.036]	0.032 ± 0.022 [0.022, 0.041]	0.018 ± 0.027 [0.007, 0.030]
Immobility time	3.2660 [3, 49.271]; 0.0290 *	0.366 ± 0.246 [0.262, 0.470]	0.535 ± 0.332 [0.386, 0.681]	0.364 ± 0.251 [0.258, 0.470]	0.562 ± 0.298 [0.436, 0.688]
Time by gas inlet	0.8066 [3, 48.648]; 0.4964	0.414 ± 0.230 [0.317, 0.512]	0.440 ± 0.357 [0.265, 0.582]	0.527 ± 0.285 [0.407, 0.647]	0.470 ± 0.385 [0.307, 0.633]
Time by tea ball	0.8013 [3, 48.647]; 0.4992	0.585 ± 0.230 [0.487, 0.682]	0.557 ± 0.356 [0.416, 0.732]	0.472 ± 0.285 [0.352, 0.592]	0.529 ± 0.387 [0.366, 0.693]

**Table 3:** Change from baseline data. F ratios and *p*-values are reported for main effects with significance set at *p* < 0.05. An asterisk (*) signifies when the *p*-value is significant. Data are reported as mean relative frequency ± standard deviation [95% CI].

Behaviors	F Ratio [df]; *p*-Value	Mean Relative Frequency ± Standard Deviation [95% CI]			
		CO_2_	Fox Urine	Isoflurane	Oxygen
Rears	4.7473 [3, 48.556]; 0.0056 *	0.019 ± 0.095 [−0.020, 0.059]	−0.025 ± 0.039 [−0.042, −0.008]	0.015 ± 0.044 [−0.003, 0.034]	−0.025 ± 0.073 [−0.057, 0.005]
Line crosses	2.3571 [3, 49.497]; 0.0830	0.024 ± 0.048 [0.003, 0.044]	−0.007 ± 0.039 [−0.024, 0.010]	0.0006 ± 0.033 [−0.013, 0.014]	−0.006 ± 0.040 [−0.023, 0.009]
Immobility time	2.6673 [3, 49.678]; 0.0578	−0.066 ± 0.365 [−0.220, 0.088]	0.145 ± 0.301 [0.011, 0.278]	0.003 ± 0.274 [−0.112, 0.119]	0.172 ± 0.341 [0.028, 0.316]
Time by gas inlet	0.0850 [3, 49.955]; 0.9679	0.002 ± 0.276 [−0.114, 0.118]	−0.033 ± 0.246 [−0.143, 0.075]	−0.021 ± 0.317 [−0.155, 0.112]	−0.032 ± 0.295 [−0.157, 0.092]
Time by tea ball	0.0810 [3, 49.955]; 0.9700	−0.002 ± 0.276 [−0.118, 0.114]	0.032 ± 0.248 [−0.077, 0.142]	0.021 ± 0.317 [−0.112, 0.155]	0.032 ± 0.296 [−0.092, 0.157]

**Table 4:** Data from the aversion-avoidance experiment. F ratios and *p* values are reported for main effects with significance set at *p* < 0.05. An asterisk (*) signifies when the *p* value is significant. Data are reported as mean (in seconds) [95% CI].

Groups	F Ratio [df]; *p*-Value	Mean (in Seconds) [95% CI]	
Forced Exposure (FE) or Naïve	6.1998 [1, 93]; 0.0146 *	FE	Naïve
		69.72 [56.0, 87.2]	103.30 [82.9, 128.6]
Treatment	6.6565 [1, 93]; 0.0115 *	CO_2_	Isoflurane
		69.42 [55.7, 86.4]	104.64 [83.3, 129.8]
Forced Exposure (FE) or Naïve interaction with Treatment	0.9614 [3, 93]; 0.3294	FE, CO_2_	FE, Isoflurane
		61.67 [45.2, 84.0]	78.14 [57.3, 106.5]
		Naïve, CO_2_	Naïve, Isoflurane
		79.25 [57.7, 108.7]	136.57 [100.1, 186.1]

## Supplementary Materials

The following are available online at https://www.mdpi.com/2076-2615/10/8/1431/s1, Table S1: Forced Exposure Timeline, Table S2: Aversion-Avoidance Timeline.
