# Supplementary material for: Isoflurane and Carbon Dioxide Elicit Similar Behavioral Responses in Rats
Source: Animals (Basel). 2020 Aug 16;10(8):1431. doi: 10.3390/ani10081431 (PMC7459795; doi:10.3390/ani10081431)
Supplement: Supplementary file 1 [file animals-10-01431-s001.pdf]

animals

Supplementary Files: Isoflurane and Carbon Dioxide Elicit Similar Behavioral Responses in Rats

Satyajit Kulkarni and Debra Hickman *

Table S1. Forced Exposure Timeline.

Sunday	Monday	Tuesday	Wednesday	Thursday	Friday	Saturday
(Dec 15)	(Dec 16)	(Dec 17)	(Dec 18)	(Dec 19) Forced Exposure Day 1	(Dec 20) Forced Exposure Day 2	(Dec 21) Forced Exposure Day 3
(Dec 22) Forced Exposure Day 4	(Dec 23)	(Dec 24)	(Dec 25)	(Dec 26) Forced Exposure Re-exposure Day 1	(Dec 27) Forced Exposure Re-exposure Day 2	(Dec 28) Forced Exposure Re-exposure Day 3
(Dec 29) Forced Exposure Re-exposure Day 4						

Table S2. Aversion-Avoidance Timeline

Sunday	Monday	Tuesday	Wednesday	Thursday	Friday	Saturday
(Jan 5)<br><br>Acclimate Group A (day 1) If going well, start group B (day 1)	(Jan 6)<br><br>Acclimate Group A (day 2) Acclimate Group B (day 2)	(Jan 7)<br><br>Test Group A (Day 1 isoflurane) Test Group B (Day 1 isoflurane) Acclimate Group C	(Jan 8)<br><br>Test Group A (Day 2 isoflurane) Test Group B (day 2 isoflurane) Acclimate Group C	(Jan 9)<br><br>Test Group B (day 1 CO2) Test Group C (day 1 CO2) Acclimate Group D	(Jan 10)<br><br>Test Group B (day 2 CO2) Test Group C (day 2 CO2) Acclimate Group D	(Jan 11)<br><br>Test Group D (day 1 isoflurane) Acclimate Group E
(Jan 12)<br><br>Test Group D (day 2 isoflurane) Acclimate Group E	(Jan 13)<br><br>Test group E (day 1 CO2) Don’t acclimate anyone today.	(Jan 14)<br><br>Test group E (day 2 CO2) Don’t acclimate anyone today	(Jan 15)<br><br>Test group E (day 1 isoflurane) Acclimate Group F	(Jan 16)<br><br>Test group E (day 2 isoflurane) Acclimate Group F	(Jan 17)<br><br>Test Group F (day 1 CO2) Acclimate Group G & H	(Jan 18)<br><br>Test group F (day 2 CO2) Acclimate Group G &H
(Jan 19)<br><br>Test Group G (day 1 isoflurane) Test Group H (day 1 isoflurane)	(Jan 20)<br><br>Test Group G (day 2 isoflurane) Test Group H (day 2 isoflurane)	(Jan 21)<br><br>Test Group H (CO2 day 1)	(Jan 22)<br><br>Test Group H (CO2 day 2)			

Group A: 2 naïve rats (isoflurane); Group B: 4 FE rats (iso first 2 days, CO2 second 2 days); Group C: 6 naïve rats (CO2); Group D: 6 naïve rats (isoflurane); Group E: 6 FE rats (CO2 first 2 days, isoflurane second 2 days); Group F: 6 naïve rats (CO2); Group G: 4 naïve rats (isoflurane); Group H: 2 FE rats (iso first 2 days, CO2 second 2 days).

© 2020 by the authors. Licensee MDPI, Basel, Switzerland. This article is an open access article distributed under the terms and conditions of the Creative Commons Attribution (CC BY) license (http://creativecommons.org/licenses/by/4.0/).
